# Improving Communication and Teamwork Between Resident Doctor Teams Using a Group Messaging Application: A Quality Improvement Project

**DOI:** 10.7759/cureus.77722

**Published:** 2025-01-20

**Authors:** Borna A Assarian, Muhammed Shahid, Melika Z Assarian, Erisa Ito

**Affiliations:** 1 Geriatrics, University Hospital Southampton NHS Foundation Trust, Southampton, GBR; 2 Geriatrics, Brighton and Sussex Medical School, Brighton, GBR; 3 Geriatrics, Barts and The London School of Medicine and Dentistry, London, GBR

**Keywords:** communication, messaging application, patient care, quality improvement project, resident doctors, teamwork, well-being, whatsapp

## Abstract

Background

Communication and teamwork are essential in medicine. Established standards emphasise the importance of collaboration and access to support. Some departments divide doctors into teams; however, inter-team communication is not always efficient. This quality improvement project aimed to improve teamwork and communication between resident-doctor teams at the Medicine for Older People (MOP) department at University Hospital Southampton using a messaging application. Secondary objectives were to assess the management of workload, resident doctor well-being, and patient safety.

Materials and methods

A WhatsApp (Meta Platforms, Inc., Menlo Park, California, United States) communication group was created to involve all eight MOP teams (no patient information was discussed). Anonymous questionnaires were used pre-implementation and two months post-implementation to assess the impact on communication, teamwork, overtime hours, doctor well-being, and patient safety. Data analysis was carried out using the IBM SPSS Statistics for Windows, Version 30 (Released 2024; IBM Corp., Armonk, New York, United States). We calculated 95% confidence intervals (CIs) and p-values using the Wilcoxon signed-rank test.

Results

All 24 resident doctors (Foundation/Senior House Officers) participated, and 19 completed both questionnaires. Significant improvements were seen in communication (very good/good ratings: pre-implementation 5%; post-implementation 100%, 95% CI 1.62-2.58, p<0.001), teamwork (pre-implementation 26%; post-intervention 84%, 95% CI 1.12-2.15, p<0.001), and workplace well-being (pre-intervention 47%; post-intervention 84%, 95% CI 0.47-1.21, p=0.001). Mean days worked overtime per week were reduced from 1.89 to 1.31 (95% CI (-0.91) - (-0.25), p=0.005) and the mean time worked overtime was reduced from 29.7 minutes/week to 19.9 minutes/week (95% CI (-14.61) - (-4.92), p=0.003). All the doctors (100%) strongly agreed/agreed the intervention helped distribute tasks more evenly and facilitated the timely completion of work. Around 90% felt this very positively/positively impacted patient safety. All doctors recommended this intervention for future residents.

Conclusion

Group messaging applications, such as WhatsApp, can enhance communication between resident-doctor teams, leading to improvements in supportive teamwork, patient care, and doctor well-being.

## Introduction

Communication and teamwork are crucial for healthcare institutions to operate safely and efficiently. The General Medical Council (GMC) good medical practice has established standards on doctor communication and teamwork, stating that doctors “must work collaboratively with colleagues” and be “readily accessible to colleagues seeking information, advice, or support” [1].

To help meet these standards, various systems exist to facilitate communication between healthcare professionals, such as telephones, pager systems, and emails. However, some of these systems are becoming increasingly outdated with their limitations, such as inefficiency and accessibility. As technology develops, the usage of smartphones among the general public and doctors has grown. This offers opportunities for various communication platforms to be used to further aid and enhance communication.

WhatsApp (Meta Platforms, Inc., Menlo Park, California, United States) is one of the most popular free-to-download mobile messaging applications worldwide. It is available on all smartphones, enables users to send and receive end-to-end encrypted messages instantly, and has features such as creating group chats and chat-clearing functions [2]. These features highlight it as an efficient and secure platform for communication, which further emphasises the application’s benefits for communication.

This quality improvement project aimed to assess and improve communication and teamwork among doctors working in the Medicine for Older People (MOP) department at University Hospital Southampton (UHS) by introducing a WhatsApp group. Data was collected through questionnaires completed by doctors before and after the implementation of the WhatsApp group, offering insight into its effect on communication, teamwork, resident doctor well-being, and other factors.

## Materials and methods

This study was conducted within the MOP department at University Hospital Southampton, United Kingdom, using a model for improvement plan-do-study-act (PDSA) cycle methodology as a quality improvement project. The study targeted foundation year one doctor (F1s) and senior house officers (SHOs) during the rotation block spanning August to December 2023. Resident doctors not based on the MOP department (n=7), locum doctors (n=2), and the study conductor were excluded from the study, leaving 24 resident doctors eligible to participate.

An initial questionnaire was sent to the 24 resident doctors at the start of the rotation (August 2023) (Appendix 1). Two months later, a WhatsApp group was created as the intervention (October 2023), and two months after this date, a final questionnaire was sent to participants (December 2023) (Appendix 2). All the doctors who participated in the study already had the application downloaded on their smartphones. Consent was gained from all participants via the questionnaires.

The messaging group was created using WhatsApp with an automated regular chat-clearing function. Doctors were invited to join the group, and an informative poster was produced and displayed in the doctor’s office. All eligible 24 doctors joined the group and were informed that patient information should not be discussed to maintain confidentiality. Participants' WhatsApp application and the WhatsApp group were recommended to be passcode protected for further protection. It was made clear that the WhatsApp group aimed to facilitate communication within the team to improve teamwork and organisation. 

Two months post-intervention (creation of WhatsApp group), participants were sent an anonymous online questionnaire to complete within two weeks to minimise recall bias. Of the 24 eligible participants, 19 (79%) completed the questionnaires. The questionnaires had five questions assessing domains of communication, teamwork, well-being, and working overtime before the intervention. Responses were given on a five-point Likert scale (Strongly Agree = 5/5, Agree = 4/5, Average = 3/5, Disagree = 2/5, and Strongly Disagree = 1/5). The post-questionnaire asked the same questions assessing the domains in the pre-questionnaire with responses given as a five-point Likert scale. Other questions assessed the amount of time resident doctors spent working overtime and their perspectives on the impact on patient safety.

The primary objective of this quality improvement project was to assess whether a group messaging intervention can have a positive effect on the communication and teamwork of resident doctors (Foundation/Senior House Officers) in the MOP department. Resident doctors were excluded if they were not based on the main wards on site or if they were locum bank doctors.

Data was analysed using the IBM SPSS Statistics for Windows, Version 30 (Released 2024; IBM Corp., Armonk, New York, United States). The Wilcoxon signed-rank test was used to determine statistical significance (p < 0.05) and 95% confidence intervals (CI).

## Results

All resident doctors participated in the WhatsApp group from implementation till the end of the placement (two months). Nineteen out of 24 doctors completed the questionnaires (79%). Results were collected and analysed. Key findings of the results are displayed in Tables 1-6.

**Table 1:** Response to communication, teamwork, and well-being domains

Questions	% responses (very good or good) before WhatsApp group creation (n)	% responses (very good or good) after WhatsApp group creation (n)	95% confidence interval, difference in responses (Likert scale 1-5)	P-value
How would you describe communication among resident doctors?	5.3% (n=1)	100% (n=19)	1.62-2.58	< 0.001
How were teamwork dynamics among resident doctors?	26.3% (n=5)	84.2% (n=16)	1.12-2.15	< 0.001
How was your overall well-being at work?	47.4% (n=9)	84.2% (n=16)	0.47-1.21	0.001

**Table 2:** Mean number of days worked overtime per week

Question	Mean number of days worked overtime per week before WhatsApp group creation	Mean number of days worked overtime per week after WhatsApp group creation	95% confidence Interval, difference in responses	P-value
On average, how many days per week did you work overtime?	1.89	1.32	(-0.91) - (- 0.25)	0.005

**Table 3:** Estimated mean minutes worked overtime on an average day worked overtime

Question	Mean minutes worked overtime on average day before WhatsApp group creation	Mean minutes worked overtime on an average day after WhatsApp group creation	95% confidence interval, difference in responses	P-value
On an average day you worked overtime, how long did you work overtime for?	29.7	19.9	(-14.61) - (-4.92)	0.003

**Table 4:** Responses to the distribution of workload

Question	% strongly agree (n)	% agree (n)	% disagree (n)	% strongly disagree (n)
Did the WhatsApp group help distribute tasks more evenly, increasing the likelihood of you finishing on time and completing your work?	78.9% (n=15)	21.2% (4)	0% (0)	0% (0)

**Table 5:** Responses to patient safety

Question	% very positive/positive (n)	% no impact (n)	% very negative/negative impact (n)
How did the WhatsApp group impact patient safety?	89.5% (n=17)	10.5% (n=2)	0% (0)

**Table 6:** Recommendation of the WhatsApp group to the next set of junior doctors

Question	% Yes (n)	% No (n)
Would you recommend the use of a resident doctor WhatsApp group to the next set of resident doctors?	100% (n=19)	0% (0)

Communication, teamwork dynamics, and wellbeing

Results from Table 1 showed domains including communication, teamwork dynamics, and overall well-being improved significantly, with all results being statistically significant. Communication was rated as very good or good by 5% of resident doctors (n=1) pre-intervention. Post-intervention, all doctors rated communication as good or very good (95% CI 1.62-2.58, p<0.001). Comments included improved communication regarding sharing workload and information, shift changes, and late arrivals. Teamwork was rated as good or very good by 26% of doctors (n=5) pre-intervention compared to 84% (n=16) post-intervention (95% CI 1.12-2.15, p<0.001). Overall well-being was rated as good or very good by 47% of doctors pre-intervention and 84% post-intervention (95% CI 0.47-1.21, p=0.001). Doctors reported that the WhatsApp group helped identify and support colleagues experiencing heavier workloads. These results show the profound impact the WhatsApp group had on teamwork between doctors.

Workload and other questions

The mean number of days working overtime per week decreased by 0.58 days from 1.89 days pre-intervention to 1.31 days post-intervention (95% CI (-0.91) - (-0.25), p=0.005) as seen in Table 2. On days working overtime, the mean time worked overtime decreased by 9.8 minutes from 29.7 minutes to 19.9 minutes (95% CI (-14.61) - (-4.92), p=0.003) as seen in Table 3. Other results are shown in Table 3-6. Uniformly, all doctors agreed or strongly agreed that the WhatsApp group helped distribute tasks more evenly and increased the likelihood of finishing work on time. Regarding patient safety, 90% of participants rated the WhatsApp group as having a positive or very positive impact on patient safety, with 10% rating no difference. This was related to the increased time doctors had to see patients due to better workload division. All doctors recommended using WhatsApp for the next set of resident doctors. All results reported p-values < 0.01, indicating that all results were highly statistically significant.

Descriptive written comments from resident doctors further highlighted the various positive impacts of the WhatsApp group; examples include: “made communication easier and faster, made teamwork and support among colleagues easier and better,” “good all round to build team spirit up and for everyone to work together well,” “received a lot more support through the group and could support other teams when they were struggling as well,” “eases communication, which makes the job and teamwork much easier,” “creates an atmosphere of teamwork,” “great communication tool” and “helped team cohesion with other residents.”

## Discussion

Patients within the MOP department are allocated by locality. Inadequate communication and teamwork between resident doctors in different localities contributed to uneven workload distribution across the localities. This resulted in some doctors having higher workloads and staying later than rostered. A high workload can lead to fatigue, burnout, and therefore increased risk of making errors, compromising patient safety [3]. Poor communication has been associated with negative patient outcomes, including loss of important information [4,5]. Using WhatsApp to communicate between the localities improved communication and teamwork between resident doctors significantly, reducing both the mean number of days working overtime per week and the mean time worked overtime on those days. All doctors agreed or strongly agreed that the WhatsApp group helped distribute tasks more evenly, and they all recommended it for the next set of rotating resident doctors. The majority felt that the improved communication via the WhatsApp group positively impacted patient safety (e.g., due to better division of workload) and the doctor’s well-being at work. 

The WhatsApp group provided an instant group messaging service that improved communication between doctors. This enabled doctors to more easily ask for support with workload, express capacity to help others, notify regarding absences or late arrivals, notify doctors about team meetings or educational sessions, and improve organisation within the department team. Resident doctors mentioned that the WhatsApp group “eases communication, which makes the job and teamwork much easier,” “creates an atmosphere of teamwork,” is a “great communication tool,” and “helped team cohesion with other residents,” reinforcing the significance of the WhatsApp group. The use of the plan-do-study-act (PDSA) method provided a systematic approach to highlight areas that required improvement and to assess the impact of the communication aid. These results were presented to the MOP department team, and given the noticeable improvements, they decided to continue the WhatsApp group for the next set of resident doctors. Administrative staff who manage the department smartphone were made admins of the group so they could remove old and add new resident doctors for each rotation as part of the induction.

Current communication amongst doctors includes email, telephone, and one-way pager systems. While having widespread use, they have their limitations. Emails provide written records of conversations and asynchronous communication; however, emails may go unnoticed due to their high volume and lack of regular use. Additionally, emails may mistakenly get filtered into spam and junk folders. Limitations of hospital pagers are well documented, including their one-way communication, frequent interruptions, and inability to differentiate between urgent and non-urgent pages, leading to difficulty prioritising tasks and reduced workflow efficiency [6-8].

A significant proportion of American doctors use smartphones at work [9]. Systematic reviews studying the impact of smartphone apps and social media on improving communication among physicians have shown their potential [10,11]. However, there is insufficient robust evidence to support this relationship. This is due to factors such as small sample sizes, limited study periods, as in this study, and differing methodologies and outcomes. Nevertheless, studies indicate that healthcare professionals prefer mobile technology over traditional methods like hospital pagers, citing improved communication efficiency [10-13]. In one study, 87% favoured the continued use of mobile technology, whilst in a randomised controlled trial (RCT), 85% recommended it for communication among healthcare professionals [12,13]. The RCT conducted by Przybylo JA et al. compared hospital pagers (control group, n=22) to a Health Insurance Portability and Accountability Act (HIPAA)-compliant group messaging (HCGM) application found on smartphones (HCGM, intervention, n=41) [13]. Satisfaction rates were significantly higher when using HCGM (p=0.003), with participants rating communication as more efficient (p=0.009) and more effective at integrating work into ward rounds (p=0.018). Compared to this study, the RCT had a larger sample size but was still a single-centre study, possibly limiting the generalisability of the results. However, results from the RCT favoured the use of smartphones in improving workload and communication, as similarly seen in this study.

Limitations of mobile phone technology within healthcare institutions must also be addressed. Introducing mobile phones can increase the frequency of messages being sent, leading to greater interruptions and an inability to distinguish between urgent and non-urgent messages [14]. These limitations can be addressed by using certain mobile applications that can reduce the effect of these factors. WhatsApp has features including the ability to organise messages into different channels, highlight certain features of messages or pin messages to indicate their importance, and mute chats to reduce interruptions. However, all these features must be used in a controlled manner to avoid introducing new issues such as missing important patient information or tasks.

Doctors, as well as the general public, are increasingly using WhatsApp due to its instant messaging feature enabling real-time, end-to-end encrypted communication. WhatsApp allows for rapid communication between doctors and allows them to reply directly to specific messages. WhatsApp also allows users to share resources in the form of images and documents, enhancing its function in educational and clinical practice. This capability, combined with features such as sending voice messages, makes the application an efficient tool for improving communication in clinical practice. Moreover, WhatsApp’s end-to-end encryption ensures the safe transfer of data between the sender and receiver [2]. As in this study, WhatsApp users can further increase security by using passcode-protected access to the application and group chat. These features support the platform’s applicability to aid communication in healthcare settings.

Multiple studies have demonstrated the effectiveness WhatsApp has in improving communication, workload productivity, and patient outcomes [15-21]. A study by Ali Astarcioglu et al. explored the use of WhatsApp to communicate between emergency physicians in rural hospitals without percutaneous coronary interventions (PCI) and tertiary hospitals with PCI capability. Results showed a significant reduction in the time taken to perform PCI in patients presenting with ST-elevated myocardial infarction (STEMI) (n=53, p<0.001) with an additional significant reduction in false STEMI rate [15]. Improvements in communication have also been seen elsewhere. Using WhatsApp to communicate between resident doctors in an oral and maxillofacial department at a major trauma centre saw improved support, communication, and teamwork similar to that seen in this study [16]. The study conducted by Dungurwalla et al. strictly only allowed discussion of work-related matters, with no messages being sent on the weekend. This allowed a boundary to be established between home and work matters and further reflected the separate communication with the weekend on-call team [16]. Within this study, there was no restriction on times and days of communication; however, participants had the option to mute or unmute the group as needed after office hours.

Improved communication and teamwork were also seen within orthopaedic surgical teams when using WhatsApp compared to traditional hospital telephones and pagers [17]. The study by Ellanti et al. was estimated to have saved at least 4790 minutes over the six-month study period when using WhatsApp compared to traditional communication devices. Emergency surgical teams saw improved communication among team members, including senior clinicians' ability to oversee team activities without direct involvement, better communication between resident doctors, and better support from senior clinicians [18]. Diagnosis of plain radiographic hand fractures using WhatsApp has also shown similar diagnostic accuracy to that of desktop computers, illustrating the broad multi-feature capabilities of WhatsApp within clinical practice [19]. The study showed fracture identification and characterisation using WhatsApp vs. desktop had good intraobserver agreement (0.60 < ƙ < 0.80) with both systems giving diagnostic accuracies above 95%. A different study involving 35 participants looked at the use of WhatsApp in improving communication within a pathology laboratory. Results showed significant improvements in awareness of critical alert reporting, duty roster, academic activities, accident/incident, and transmission of information (p<0.001) [20]. However, the results also found significant increases in workload, interruptions at work, and unproductive and misdirected spreading of information (p<0.001) with all results within statistical significance.

WhatsApp, similar to other communication platforms, has its benefits but also limitations. Sending and receiving messages requires a reliable internet connection. Some areas of hospitals may not have a strong signal and internet connection to support communication through the platform. In these cases, methods like pagers remain beneficial. The matter of breaching confidentiality is also a concern. However, having multiple security features, such as end-to-end encryption, automated chat deletion, and passcode protection, strengthens its security [2]. The implementation of WhatsApp groups is not intended to replace current existing methods like pagers but rather to aid communication alongside them.

Limitations of the study included a single speciality, single-centre study and the possibility of social desirability bias. Only resident doctors working within the department could be recruited, which meant the sample size was limited. However, all findings within communication, teamwork, well-being, and workload demonstrated significant statistical differences. The sample size can be increased if the study is conducted across multiple departments and hospitals. The long-term impact of this study is also unknown, given the intervention was carried out for a limited time of two months (half a rotation). Collecting data post-intervention over a longer time period would provide better evidence of the effectiveness of the intervention. There was also a risk of participants having recall bias when responding to the questionnaire; however, providing the two-week window for the completion of the questionnaire reduced and minimised the possibility of this. 

## Conclusions

WhatsApp is a growing telecommunication tool that, given its multi-featured, real-time, end-to-end encrypted instant messaging service, provides great potential to improve communication within team settings. In our study, we saw how a WhatsApp group significantly improved teamwork and communication between resident doctors. This had highly beneficial impacts on resident doctors' well-being by improving workload distribution and reducing overtime working hours. Participants also described enhancements in patient safety, which further emphasised the positive effects of this communication aid.

## Appendices

Appendix 1

Initial Questionnaire

Before: Medicine for Older People (MOP) quality improvement project assessing communication and teamwork between resident doctors in the MOP department and whether this impacts factors such as working overtime, well-being, and others. This is intended to be anonymous, and all efforts have been made to ensure anonymity. By completing the form, you consent to your answers being used in the study.

* Indicates required question

1. Communication*

How would you describe the communication among resident doctors? (e.g. regarding notifying of late arrivals, sickness notification, general communication, group morale/social interactions) (Mark only one)

Very good

Good

Average

Bad

Very bad

2. Please share your experience in more detail:

3. Teamwork*

How is the teamworking dynamics among resident doctors? (e.g. regarding asking for support with ward jobs/workload, appropriate management of doctor numbers per team, asking questions and advice) (Mark only one)

Very good

Good

Average

Bad

Very bad

4. Please share your experience in more detail:

5. Working overtime*

On average, how many days per week do you work overtime? (Mark only one)

0

1

2

3

4

5

6. Working overtime*

On an average day you worked overtime, how long did you work overtime for? (Mark only one)

<9 minutes

10- 19 minutes

20 -29 minutes

30- 39 minutes

40- 49 minutes

50- 59 minutes

>60 minutes

7. Please share your experience in more detail:

8. Well-being* 

How is your overall well-being at work? (e.g., sense of community within the group, access to support, assistance with finishing on time, division of workload, stress, work-life balance, general support) (Mark only one)

Very good

Good

Average

Bad

Very bad

9. Please share your experience in more detail:

Appendix 2

Final Questionnaire

After: Medicine for Older People (MOP) quality improvement project assessing communication and teamwork between resident doctors in the MOP department after the implementation of a WhatsApp group and whether this impacts factors such as working overtime, well-being, and others. This is intended to be anonymous, and all efforts have been made to ensure anonymity. By completing the form, you consent to your answers being used in the study.

* Indicates required question

1. Communication*

After implementation of the resident doctor WhatsApp group, how would you describe the communication among resident doctors? (e.g., regarding notifying of late arrivals, sickness notification, general communication, group morale/social interactions) (Mark only one)

Very good

Good

Average

Bad

Very bad

2. Please share your experience in more detail:

3. Teamwork*

After implementation of the resident doctor WhatsApp group, how was the teamworking dynamics among resident doctors? (e.g. regarding asking for support with ward jobs/workload, appropriate management of doctor numbers per team, asking questions and advice) (Mark only one)

Very good

Good

Average

Bad

Very bad

4. Please share your experience in more detail:

5. Working overtime*

After the implementation of the WhatsApp group, on average, how many days per week did you work overtime? (Mark only one) 

0

1

2

3

4

5

6. Working overtime*

After implementation of the WhatsApp group, on an average day you worked overtime, how long did you work overtime for? (Mark only one)

<9 minutes

10- 19 minutes

20 -29 minutes

30- 39 minutes

40- 49 minutes

50- 59 minutes

>60 minutes

7. Please share your experience in more detail:

8. Working overtime*

Did the WhatsApp group help distribute tasks more evenly, increasing the likelihood of you finishing on time and completing your work? (Mark only one)

Strongly agree

Agree

Disagree

Strongly disagree

9. Well-being*

After the implementation of the resident doctor WhatsApp group, how was your overall well-being at work? (e.g., sense of community within the group, access to support, assistance with finishing on time, division of workload, stress, work-life balance, general support) (Mark only one)

Very good

Good

Average

Bad

Very bad

10. Please share your experience in more detail:

11. Patient safety*

How did the WhatsApp group impact patient safety? (e.g., regarding division of workload to complete tasks, time for patient assessments/consultations, accessibility to ask for support to manage patient loads) (Mark only one)

Very positive

Positive

No difference

Negative

Very negative

12.Please share your experience in more detail:

13. Future*

Would you recommend the use of a resident doctor WhatsApp group to the next set of resident doctors coming to MOP? (Mark only one)

Yes

No

14. Please explain why you selected your answer:*

15. Please share any other comments or suggestions in regard to improving communication, teamwork, working overtime, patient safety, and resident doctor well-being in the MOP rotation:
